# Minor Cytological Abnormalities and up to 7-Year Risk for Subsequent High-Grade Lesions by HPV Type

**DOI:** 10.1371/journal.pone.0127444

**Published:** 2015-06-17

**Authors:** Maria Persson, K. Miriam Elfström, Sven-Erik Olsson, Joakim Dillner, Sonia Andersson

**Affiliations:** 1 Department of Women’s and Children’s Health, Division of Obstetrics and Gynecology, Karolinska Institutet, Elevhemmet H2:00, Karolinska University Hospital Solna, Stockholm, Sweden; 2 Department of Medical Epidemiology and Biostatistics, Karolinska Institutet, Stockholm, Sweden; 3 Department of Clinical Sciences, Danderyd’s Hospital AB, Karolinska Institutet, Division of Obstetrics and Gynecology, Stockholm, Sweden; Penn State University School of Medicine, UNITED STATES

## Abstract

**Objective:**

Diagnoses of atypical squamous cells of undetermined significance (ASCUS) and low-grade squamous intraepithelial lesions (LSIL) are common, but the corresponding risk of disease varies by human papillomavirus (HPV) status, complicating management strategies. Our aim was to estimate the longer-term risk of cervical intraepithelial neoplasia grade 2 or worse (CIN2+) among women with ASCUS/LSIL by age, HPV status, and genotype(s).

**Methods:**

A total of 314 women with ASCUS/ LSIL were followed for a median of 3.8 years. Baseline HPV status was determined by reflex testing and women with histologically confirmed CIN2+ were identified through linkage to the Swedish National Quality Register for Cervical Cancer Prevention. Cumulative incidence and hazard ratios were estimated to explore differences between index data and associations with CIN2+.

**Results:**

In total, 89 women (28.3%) developed CIN2+. High-risk (HR) HPV-positive women developed significantly more CIN2+ than HR-HPV-negative women (cumulative incidence 3.5 years after the index test: 42.2%, 95% CI: 32.5–53.5 for HPV16/18; 36.2%, 95% CI: 28.3–45.4 for other HR-HPV types; and 2.0%, 95% CI: 0.5–7.8 for HR-HPV-negative women; p<0.0001).

**Conclusion:**

HPV status was of greatest importance in determining the risk of CIN2+. The risk was low among HPV-negative women during the first years of follow-up, suggesting these women could be followed less intensively. HPV16/18-positive women may need intensified follow-up as they showed the highest risk of CIN2+.

## Introduction

Screening programs based on cervical cytology have significantly reduced the incidence and mortality of cervical cancer since their introduction in the 1960s [1–3]. However, cytology testing has variable sensitivity and reproducibility in detecting precancerous lesions, cervical intraepithelial neoplasia (CIN) [4]. An appealing alternative is to test for viral infection caused by high-risk human papillomavirus (HR HPV) [5].

Most HPV infections regress spontaneously and only a minority become persistent. However, these persistent infections carry a substantial risk for progression to CIN, which may progress further to invasive cervical cancer [6–8]. The risk of progression to cancer also varies between different HR HPV genotypes and the strongest risk is associated with HPV16, the most common genotype [9]. HPV18 is the second most common genotype in squamous cervical cancer and is particularly associated with adenocarcinoma or its precursor, adenocarcinoma in situ [10]. In contrast to squamous cervical cancer, incidence of adenocarcinoma continues to increase in developed countries despite cervical cytology screening program efforts [11]. HPV16 and18 are found in approximately 70% of squamous cervical cancer [12, 13].

The majority of cytological abnormalities detected in the Swedish cervical screening program are of minor grade. Of all cervical samples taken in 2012, 5.74% had a low-grade abnormality (atypical squamous cells of undetermined significance (ASCUS, 3.61%) or low-grade intraepithelial lesions (LSIL, 2.13%)) [14]. Many studies have shown that the risk of developing pre-cancer and invasive cervical cancer is very low if the woman is HPV negative [15–17]. Therefore triaging ASCUS cytology results with HPV DNA testing is a recommended strategy to identify women who need referral for colposcopy but also to provide reassurance to women who are HPV negative [5].

Swedish guidelines now recommend HPV triage of all women with minor cytological abnormalities regardless of ASCUS or LSIL diagnosis. HPV-positive women are referred for colposcopy and HPV negative women are recommended to undergo repeat cytology testing one year later, for safety reasons [18,19]. In recent years, there has been an increase in diagnoses of minor cytological abnormalities, resulting in increased colposcopy referrals. As referrals require significant time and resources on the part of the health care system and can be stressful for women, this raises the question of whether HPV negatives could be referred back to the three-year screening interval in the organised program.

In this study, we aimed to estimate the longer-term risk of developing cervical pre-cancer by ASCUS/LSIL status, age, and HPV DNA genotyping over up to 7 years of follow-up in order to provide insight into the management of women with minor cytological abnormalities.

## Materials and Methods

### Study population and follow-up

In 2005, a study comparing conventional cytology and liquid-based cytology (LBC) with supplementary HPV-triage was started and recruited women attending organized cervical cancer screening at 6 maternity health centres in southern Stockholm, Sweden [20]. Women were screened with conventional cytology or LBC according to the week of their appointment. Among those screened with LBC, a total of 326 women with minor cytological abnormalities, were identified and form the study base of this analysis. To be included in this longitudinal analysis and contribute to follow-up time, women had to have at least one follow-up test (cytology or histology) taken after study-entry. One woman was excluded who only had an index cytology result and no follow-up tests. Furthermore, 11 women diagnosed with a CIN2 or worse (CIN2+) lesion on the same day as the ASCUS/LSIL index cytology were also excluded, giving a final study population of 314 women. Of the remaining 314 women with minor cytological abnormalities, 76 (24.2%) had a cytological diagnosis of ASCUS and 238 (75.8%) had LSIL.

Since this study was nested within the organized screening program, women were followed according to the established clinical practice policies in Stockholm.Women with cytological abnormalities using ASCUS as a cut-off were referred for gynaecological follow-up including a pelvic examination, colposcopy with directed biopsies of suspicious areas, regardless of HPV test, HPV detection and genotyping was carried out as reflex testing of the LBC samples. Women were treated if the investigation revealed CIN2+ or persistence of a minor lesion.

### Cytology and HPV DNA testing

LBC samples collected in Preserve Cyt medium (Hologic, Bedford, MA) were obtained from all women in the study at study-entry [20]. Cytological classification was based on the 2001 Bethesda nomenclature system [21]. According to the cytological diagnostic system used, koilocytosis without signs of dysplasia was reported as a non-pathologic finding (however, koilocytosis is rare).

HPV-DNA detection and genotyping was performed using the Linear Array Genotyping Test (LA; Roche Diagnostics, Pleasanton, California, USA) on nucleic acid extracted by the MagNA Pure LC robot (Roche Diagnostics) as previously described [22]. The LA test identifies 37 different HPV types. A sample was categorized high-risk HPV (HR HPV) positive if one of the following 13 HPV types were included; 16, 18, 31, 33, 35, 39, 45, 51, 52, 56, 58, 59, 68 [23]. The Linear Array test was used in the first research studies examining the use of HPV testing in the screening program in Sweden.

All laboratory analyses were performed at the Department of Virology, Huddinge, Karolinska Hospital, Stockholm, Sweden.

### Statistical analysis

In this prospective cohort study, we included women with minor cytological abnormalities and complete HPV testing at baseline and at least one follow-up cytological or histological test. Follow-up time was calculated from the date of the index ASCUS/LSIL to the 1^st^ occurrence of a histologically confirmed CIN2+ lesion or the last registered cytology date if the individual did not develop a CIN2+ lesion.

The women were linked, using personal identification numbers, to the population-based the Swedish National Cervical Screening Registry (NKCx) to identify those individuals who developed histologically confirmed CIN2+ during follow-up, until December 31^st^ 2012. NKCx contains a copy of the same file that is used to report the cytological and histopathological diagnoses from all cytological and histopathological laboratories in Sweden. The completeness of NKCx is therefore 100% and all events occurring in the 7 years of linkage to the registry were captured. All cytological smears, histopathologies, and visits to hospitals in Sweden are registered using a Personal Identification Number that is unique for each individual and is assigned at birth or at immigration to Sweden. As we used linkages based on the PIN to a complete registry, the current study has nationwide information on all follow-up smears and histopathologies taken from all women in the studied cohort.

Firstly, we estimated the cumulative incidence of CIN2+ by baseline characteristics (age, cytological diagnosis, and HPV status) using one minus the Kaplan-Meier curves with 95% confidence intervals based on the Greenwood formula. Differences between groups were assessed using the log-rank test. Cumulative incidence were reported at 3.5 years and 6 years to reflect the estimated risk after the first and second subsequent screening round. Secondly, univariate and multivariate Cox regression models were used to show the associations between index visit data and the outcome (CIN2+ in histopathology). Hazard ratios (HR) and their 95% confidence intervals (95% CI) were obtained from the regression models using time since baseline ASCUS/LSIL result as the underlying timescale. The proportional hazards assumption was checked and no evidence of non-proportionality was found.

A p-value of <0.05 was considered statistically significant and all analyses were done using STATA version 13 (Stata Corp. Stata Statistical Software, USA).

### Ethics

Written informed consent was obtained from all study participants before enrolment and ethical permissions for the study was approved by the Regional Board of the Karolinska Institutet in Stockholm, Sweden in 2004 with updates in 2010 and 2013 (No. 04-679/3, No. 2010/944-32 and 2013/763-32).

## Results

### Characteristics of the study women

In total, 39.5% of women with ASCUS and 79.4% of women with LSILs were HR HPV positive. Overall, 214 (68.2%) women were positive for any of the 13 high-risk (HR) HPV types defined as oncogenic by the International Agency for Research on Cancer [13] but most infections were due to HPV type 16 and/or 18 (27.7%). Sixty-six women (21.0%) were HPV 16 positive and 27 (8.6%) HPV 18 positive. Of the 66 HPV 16 positive women, 8 (12.2%) were ASCUS and 58 (87.9%) were LSIL and of the 27 HPV 18 positive women, 4 (14.8%) were ASCUS and 23 (85.2%) were LSIL. The second most common HPV type in the group was HPV 51 (10.8%). Non-HR type positivity in the study population was 44.6% and 26.8% of women had HPV infections with 2 or more types. The mean age in the study group was 34 years (median 32 years, range 23–60 years) but most women (58.6%) were older than 30 years. The median follow-up time was 3.8 years (range 0.1–7.0 years, 27.3% had a follow-up time of more than 5 years). A total of 89 (28.3%) women developed histologically confirmed CIN2+ during follow-up (Table 1).

**Table 1:** Distribution of background characteristics among 314 women with ASCUS/LSIL index cytologies.

	Study population	Count of CIN2+
	N (%)	N
**Age (range: 23–60)**		
≤29	130 (41.4)	35
30–39	97 (30.9)	36
40–49	71 (22.6)	15
≥50	16 (5.1)	3
**Baseline cytology result**		
ASCUS	76 (24.2)	11
LSIL	238 (75.8)	78
**HPV status**		
HR HPV positive* (13 types)	214 (68.2)	85
HPV 16 and/or 18 positive	88 (28.0)	40
Other HR HPV positive**	127 (40.5)	46
Non-HR HPV positive***	140 (44.6)	44
**HR type co-infections**		
No HR HPV infection	100 (31.9)	4
1 HR HPV infection	130 (41.4)	53
≥2 HR HPV infections	84 (26.8)	32
**HPV type-specific positivity**		
HPV 16	66 (21.0)	33
HPV 18	27 (8.6)	12
HPV 31	33 (10.5)	20
HPV 33	15 (4.8)	10
HPV 35	14 (4.5)	4
HPV 39	22 (7.0)	9
HPV 45	21 (6.7)	8
HPV 51	34 (10.8)	9
HPV 52	31 (9.9)	7
HPV 56	28 (8.9)	8
HPV 58	18 (5.7)	7
HPV 59	20 (6.4)	7
HPV 68	10 (3.2)	2

*13 HR types: 16, 18, 31, 33, 35, 39, 45, 51, 52, 56, 58, 59, 68

**Excluding types 16 and 18

***24 non-HR types: 6, 11, 26, 40, 42, 53, 54, 55, 61, 62, 64, 66, 67, 69, 70, 71, 72, 73 (MM9), 81, 82 (MM4), 83 (MM7), 84 (MM8), IS39, and CP6108.

ASCUS: atypical squamous cells of undetermined significance, LSIL: low-grade squamous intraepithelial lesions, HPV: human papillomavirus.

### Cumulative incidence of CIN2+ by background characteristics

**Fig 1:**
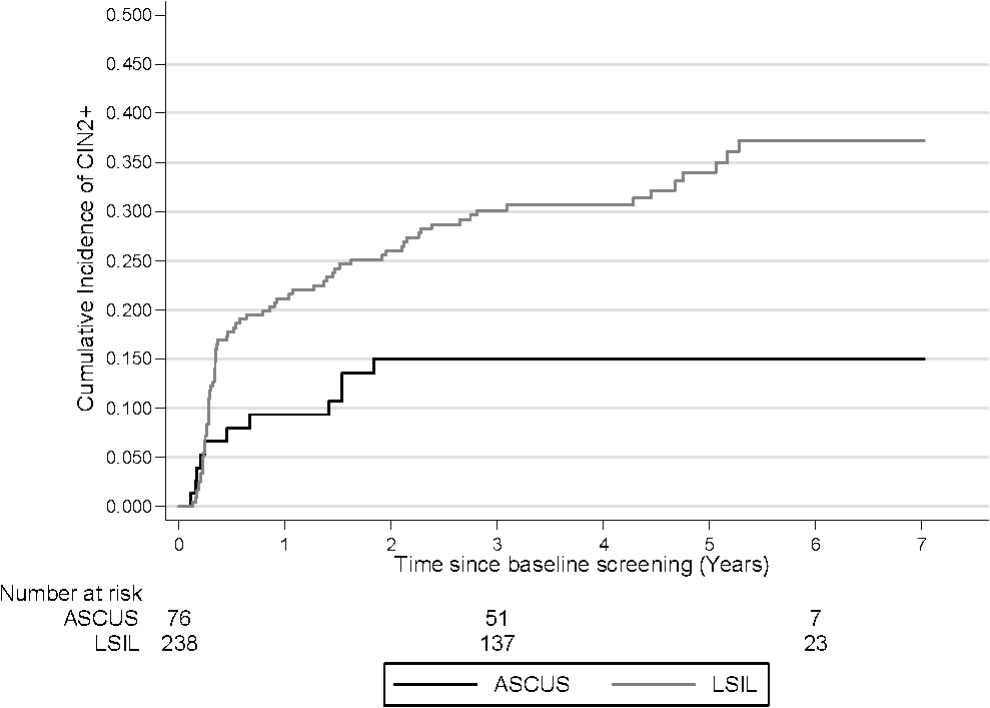
Cumulative incidence of CIN2+ by baseline test result. Log rank p-value: 0.0049 CIN 1+: cervical intraepithelial neoplasia grade 2 or worse, ASCUS: atypical squamous cells of undetermined significance, LSIL: low-grade squamous intraepithelial lesions.

**Fig 2:**
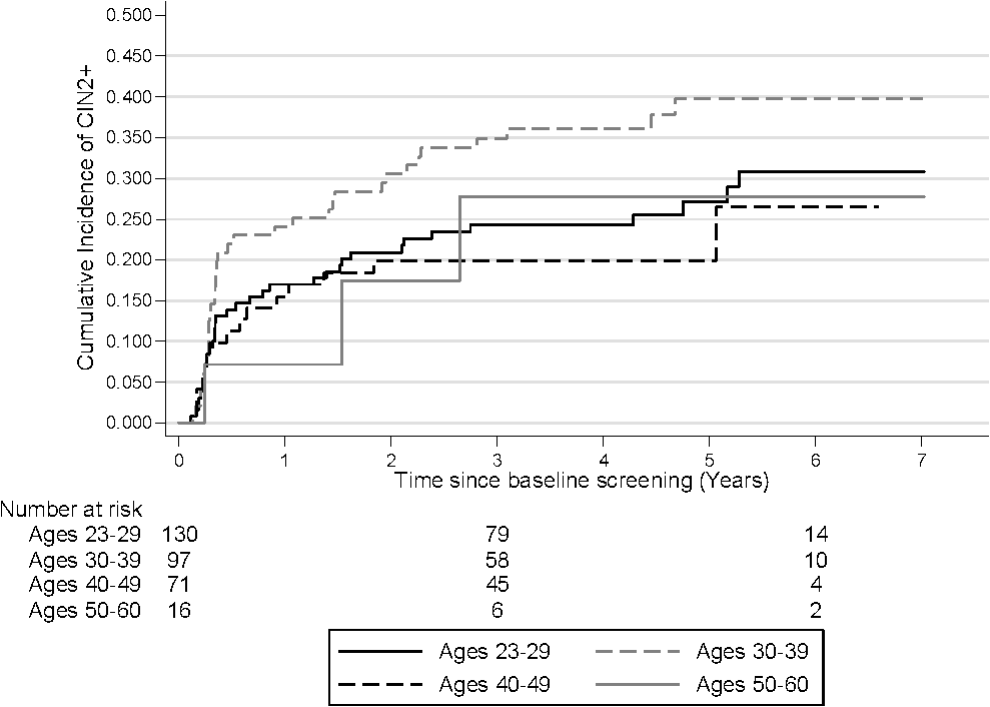
Cumulative incidence of CIN2+ by baseline age. Log rank p-value: 0.1499 CIN2+: cervical intraepithelial neoplasia grade 2 or worse

In the first two years of follow-up the cumulative incidence increased quickly for women with a baseline ASCUS result and then plateaued after two years. However, in the LSIL group, the cumulative incidence increased steadily over the entire follow-up period. The 3.5 year cumulative incidence of CIN2+ for women with a baseline ASCUS or LSIL cytology were 15.0% (95% CI: 8.6–25.5) and 30.7% (95% CI: 25.2–37.1), respectively. The overall difference between the curves was statistically significant (p = 0.0049) (Fig 1). The 3.5 year cumulative risk of developing CIN2+ was greatest among women aged 30–39 years (34.9% (95% CI: 26.2–45.4)). Among women younger than 29, the 3.5 year cumulative risk was 24.3% (95% CI: 17.8–32.8). At 6 years, these differences remained and the cumulative incidence was 30.8% (95% CI: 22.6–41.0) and 39.8% (95% CI: 30.3–51.0) for women ages 23–29 and 30–39, respectively (Fig 2).

**Fig 3:**
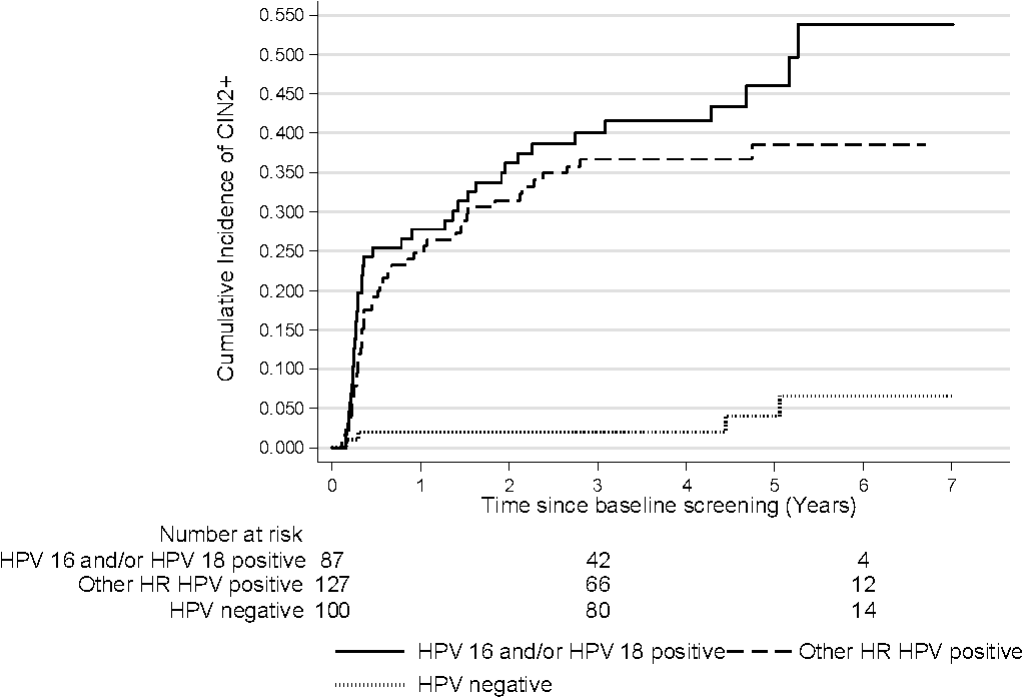
Cumulative incidence of CIN2+ by baseline HR HPV status. CIN2+: cervical intraepithelial neoplasia grade 2 or worse, HR HPV: high-risk human papillomavirus. Log rank p-value: <0.0001

HR HPV positive women had a higher cumulative risk of CIN2+ 3.5 after the index test compared to HR HPV negative women: cumulative 42.2% (95% CI: 32.5–53.5) for HPV16/18 and 36.2% (95% CI: 28.3–45.4) for other HR HPV types compared to 2.0% (95% CI: 0.5–7.8) for HR HPV negatives. Again, these differences remained after 6 years of follow-up (cumulative incidence 54.4% (95% CI: 41.2–68.7) for HPV 16/18 and 38.0% (95% CI: 29.7–47.7) for other HR HPV types compared to 6.6% (95% CI: 2.3–18.1) for HPV negatives). The overall difference between the curves was statistically significant, p<0.0001. After 5 years of follow-up, the risk for CIN2+ continued to increase among women who were HPV16/18 positive at baseline while the risk among women positive for other HR types did not increase after 5 years (Fig 3).

**Fig 4:**
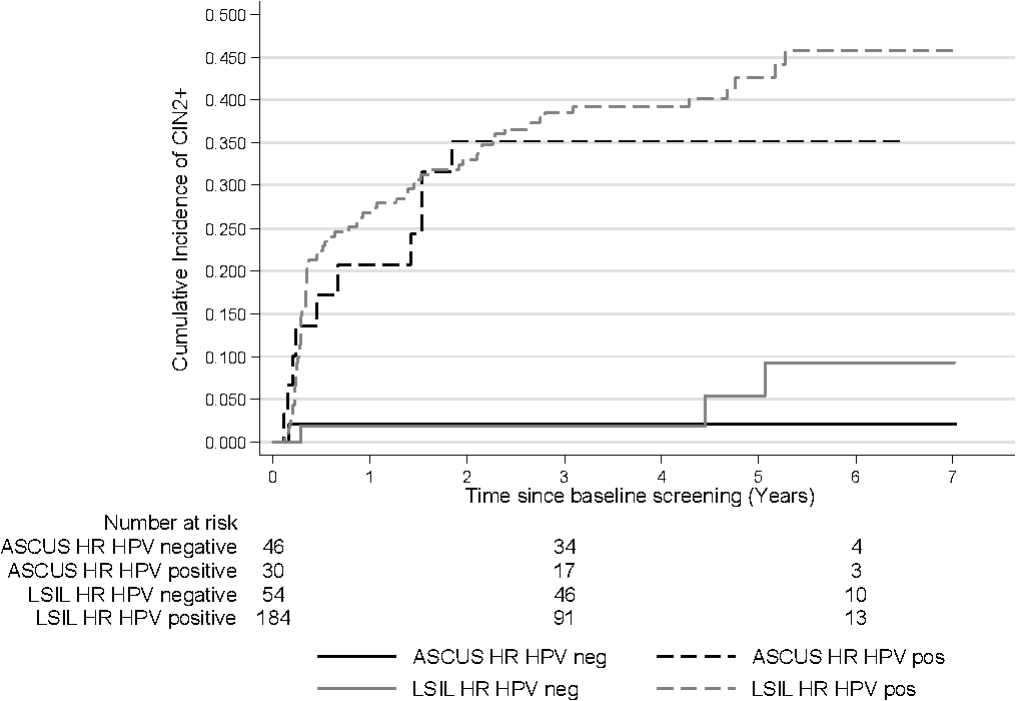
Cumulative incidence of CIN2+ by baseline test result and HR HPV status. CIN2+: cervical intraepithelial neoplasia grade 2 or worse, ASCUS: atypical squamous cells of undetermined significance, LSIL: low-grade squamous intraepithelial lesions, HR HPV: high-risk human papillomavirus. Log rank p-value: <0.0001

Overall, HR HPV positive ASCUS/LSIL had a higher cumulative risk of CIN2+ compared to HR HPV negative ASCUS/LSIL. The cumulative incidence of CIN2+ for women with HR HPV positive ASCUS was 35.2% (95% CI: 20.7–55.6) at 3.5 and 6 years of follow-up and the risk for women with HR HPV positive LSIL was 39.2% (95% CI: 32.5–46.9) at 3.5 years and rose to 45.8% (95% CI: 37.7–54.6) at 6 years of follow-up. The cumulative incidence of CIN2+ increased rapidly among women positive for HR HPV at baseline and plateaued after 2 years among women with ASCUS but continued to increase among women with LSIL. The cumulative incidence of CIN2+ for HR HPV negative women with ASCUS/LSIL remained similarly low during the first years with only a few more cases among the LSILs after 4.5 years of follow-up. The difference between ASCUS and LSIL when stratified by HPV status was statistically significant, p<0.0001. The 3.5 year risk of CIN2+ among HR HPV negative ASCUS and LSIL was 2.2% (95% CI: 0.3–14.5) and 1.9% (95% CI: 0.3–12.7), respectively. The 6-year risk among HR HPV negative ASCUS remained 2.2% but increased to 9.3% (95% CI: 3.0–27.4) for HR HPV negative LSILs (Fig 4).

**Fig 5:**
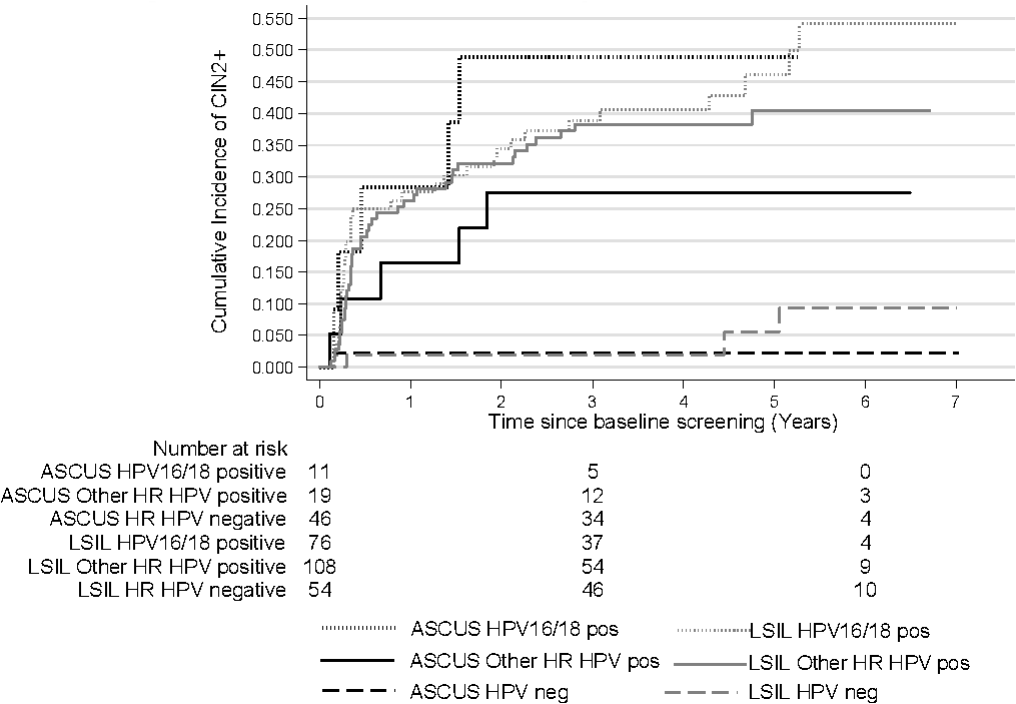
Cumulative incidence of CIN2+ by baseline test result and HPV status. CIN2+: cervical intraepithelial neoplasia grade 2 or worse, ASCUS: atypical squamous cells of undetermined significance, LSIL: low-grade squamous intraepithelial lesions, HR HPV: high-risk human papillomavirus. Log rank p-value: <0.0001

Fig 5 shows the cumulative incidence of CIN2+ among ASCUS and LSILs stratified by HPV 16/18, other HR HPV types and HPV-negativity. The highest cumulative risk of CIN2+ was observed among women positive for HPV16/18. The cumulative incidence for HPV16/18 positive ASCUS was 48.9% (95% CI: 23.8–80.8) at 3.5 years of follow-up and for HPV 16/18 LSIL it was 40.6% (95% CI: 30.3–52.8). The cumulative incidence of CIN2+ for women with ASCUS, positive for other HR HPV types was 27.6% (95% CI: 12.5–54.1) and for LSIL, positive for other HR HPV types was 38.3% (95% CI: 29.7–48.4) at 3.5 years of follow-up. The risk for CIN2+ increased rapidly among HPV positive ASCUS/LSIL during the first two years and continued to increase during the whole follow-up time. However, the risk among HPV16/18 positives was always greater than the risk among women positive for other HR HPV types (Fig 5).

### Association between baseline characteristics and CIN2+

We evaluated age, cytological, and viral risk factors for developing CIN2+ in the study group. Unadjusted associations are shown in Table 2 and adjusted associations derived from a multivariate Cox regression model are shown in Table 3. Risk factors at baseline, which were significantly associated with developing CIN2+, were women with LSIL cytology (HR 2.4; 95% CI, 1.3–4.5) compared to ASCUS and HR HPV positivity (HR 12.5; 95% CI, 4.6–34.1) compared to HR HPV negativity. Being positive in HPV genotype 16 and/or 18 (HR 2.4; 95% CI, 1.6–3.7), HPV16 alone (HR 2.6; 95% CI, 1.7–4.1), or one of the HPV types 31 (HR 3.2; 95% CI, 2.0–5.3) or 33 (HR 4.1; 95% CI, 2.1–7.9) were also significantly associated with developing CIN2+ compared to being negative for that type. Being positive HPV18 alone was borderline significant (HR 1.9; 95% CI, 1.0–3.4). Non-HR HPV positive compared to non-HR HPV negative was not significantly associated with developing CIN2+ (HR 1.2; 95% CI: 0.8–1.9). The magnitude of the association for infection with 1 HR HPV type and infection with 2 or more HPV types and CIN2+ was similar to that of infection with any HR HPV type (Table 2). In the multivariable model adjusted for age at baseline, index cytology and HPV status, the strength of association was greatest for HR HPV positivity compared to negativity (HR HPV16/18 2.3 (95% CI; 1.5–3.5) (Table 3).

**Table 2:** Unadjusted association between baseline characteristics and CIN2+.

Effect	HR (95% CI)	p-value
**Age**		
30–39 vs. ≤29	1.5 (0.9–2.4)	0.10
40–49 vs. ≤29	0.8 (0.4–1.5)	0.47
≥50 vs. ≤29	0.8 (0.3–2.7)	0.76
**Baseline cytology**		
LSIL vs. ASCUS	2.4 (1.3–4.5)	0.01
**HPV status**		
HR HPV positive* vs. HR HPV negative	12.5 (4.6–34.1)	<0.01
HPV 16 and/or HPV18 positive vs. HPV 16 and HPV 18 negative	2.4 (1.6–3.7)	<0.01
Other HR HPV positive** vs. Other HR HPV negative	1.7 (1.1–2.6)	0.01
Non-HR HPV positive vs. Non-HR HPV negative	1.2 (0.8–1.9)	0.34
1 HR HPV infection vs. No HR HPV infection	12.9 (4.7–35.8)	<0.01
≥2 HR HPV infections vs. No HR HPV infection	11.8 (4.2–33.5)	<0.01
HPV 16 positive vs. HPV 16 negative	2.6 (1.7–4.1)	<0.01
HPV 18 positive vs. HPV 18 negative	1.9 (1.0–3.4)	0.04
HPV 31 positive vs. HPV 31 negative	3.2 (2.0–5.3)	<0.01
HPV 33 positive vs. HPV 33 negative	4.1 (2.1–7.9)	<0.01
HPV 35 positive vs. HPV 35 negative	1.4 (0.6–3.5)	0.43
HPV 39 positive vs. HPV 39 negative	1.8 (0.9–3.5)	0.11
HPV 45 positive vs. HPV 45 negative	1.5 (0.7–3.0)	0.31
HPV 51 positive vs. HPV 51 negative	0.9 (0.4–1.8)	0.75
HPV 52 positive vs. HPV 52 negative	0.7 (0.3–1.5)	0.39
HPV 56 positive vs. HPV 56 negative	1.0 (0.5–2.1)	0.99
HPV 58 positive vs. HPV 58 negative	1.6 (0.7–3.5)	0.23
HPV 59 positive vs. HPV 59 negative	1.2 (0.5–2.5)	0.71
HPV 68 positive vs. HPV 68 negative	0.7 (0.2–2.7)	0.58

*13 HR types: 16, 18, 31, 33, 35, 39, 45, 51, 52, 56, 58, 59, 68

**Excluding types 16 and 18

CIN2+: cervical intraepithelial neoplasia grade 2 or worse, ASCUS: atypical squamous cells of undetermined significance, LSIL: low-grade squamous intraepithelial lesions, HR HPV: high-risk human papillomavirus.

**Table 3:** Adjusted multivariable model*—full study population.

Effect	HR (95% CI)	p-value
**Age**		
30–39 vs. ≤29	1.8 (1.1–2.8)	0.02
40–49 vs. ≤29	1.2 (0.6–2.2)	0.61
≥50 vs. ≤29	1.0 (0.3–3.4)	0.95
**Baseline cytology**		
LSIL vs. ASCUS	2.1 (1.1–4.0)	0.03
**HPV status**		
HPV 16 and/or HPV18 positive vs. HPV 16 and HPV 18 negative	2.3 (1.5–3.6)	<0.01

*All variables in the table were included as covariates.

LSIL: low-grade squamous intraepithelial lesions, ASCUS: atypical squamous cells of undetermined significance, HPV: human papillomavirus.

## Discussion

Among women with minor cytological abnormalities where we had a complete, nationwide data linkage for up to 7 years, we identified the long-term risk of developing high-grade cervical disease stratified by age, cytological, and viral risk factors. HPV status was the most significant determinant of developing high-grade cervical disease during follow-up. The long-term risk of CIN2+ following a negative HR HPV DNA test, regardless of cytological diagnosis of ASCUS or LSIL, was low during follow-up. For HR HPV-negative women with an LSIL index smear, the risk increased after 4.5 years of follow-up, suggesting that these women should be followed-up after a shorter interval than 4.5 years.

Swedish triage guidelines do not distinguish between ASCUS/LSIL diagnosis and, for safety reasons, recommend retesting at one year despite a HR HPV negative test result [19]. As our results demonstrate, there is a steady low risk of CIN2+ for HPV negative minor cytological abnormalities during the first 4.5 years of follow-up, suggesting that the re-testing after 1 year could be reconsidered (based on the performance of the HPV test used in the present study). However, given the width of the confidence intervals around the cumulative risk among HPV negative ASCUS/LSIL women, caution should be given to re-defining the interval and this analysis should be repeated in larger material to confirm the findings. Women diagnosed with HPV16/18 at baseline had the highest risk of developing CIN2+ compared to HPV negative women. Our data further show that the risk associated with being HPV 16/18 continued to increase over follow-up, even after 5 years, compared to those who were positive for other high-risk HPV types at baseline. However, the risk of developing CIN2+ was substantial for those positive for other non-16/18 HR HPV types.

The risk stratification concept introduced by Castle et al. which involves “equal management of equal risks” [24, 25] states that it is safe to return the patient to the regular screening if the 5-year risk of CIN3+ is below 2%. If the risk is between 2–10%, follow-up at one year is recommended and a risk >10% referral to colposcopy is needed [24]. Kocken et al. concluded in a study of long-term follow-up of women with borderline and mild dyskaryosis that HR-HPV negative women might be referred to routine screening, as their 5-year CIN3+ risk is negligible [26].

Risk stratification by genotyping in this group will probably not alter clinical management anyway because the risk of pre-cancer is so high that it will require immediate colposcopy. However, in populations with limited access to follow-up it might motivate immediate treatment. Commercially available HPV DNA tests that simultaneously detect HPV16 and HPV18 individually and 12 pooled HR HPV types could be beneficial [27]. The rather high risk of developing CIN2+ for LSILs reflects the high prevalence of HR HPV (79.4%) in this diagnostic category. As in our study, several others have previously demonstrated high risks for pre-cancer among HPV positives and especially among women positive for HPV 16 and/or 18 [26,28,29].

The low risk of HPV negative ASCUS has been confirmed in a large cohort study by Katki et al. [18]. They concluded that women with HPV-negative ASCUS had a similar 5-year risk for CIN2+/CIN3+ as women with a normal Pap smear and therefore could be managed similarly, namely with a 3-year retesting interval. In the same cohort, Katki et al. [30] also studied the risk of LSILs developing high-grade cervical disease and found that the HPV-test result clearly modified the risk but only when considering repeat testing of HPV negative LSILs at one year rather than immediate colposcopy [31]. Several large randomised controlled studies and longitudinal cohort studies have demonstrated that HPV negativity is protective against development of pre-cancer [15,16,32] but 100% reassurance can never be guaranteed. Castle et al made a review of case histories CIN3 cases that were HR HPV negative at baseline and found evidence that these cases were due to incident (new) cases, non HR HPV, misclassified histology and false negative HR HPV. They concluded that among women with cytological abnormalities, there will be a few cases of cervical pre-cancer that will test HR HPV negative for one or more reasons [33]. As our data also illustrates, there is a small, but not negligible, long-term risk of pre-cancer among HPV negative women.

A recent study from the Norwegian Cervical Cancer Screening Programme compared the short-time (6 months) and long-time (3 years) effectiveness of different HPV tests and reported that the risk for CIN2+ in HPV-negative women with persistent ASCUS/LSIL was over 2% and that return these women to the normal screening was potentially unsafe. However, a normal repeat cytology at 6 months following an ASCUS/LSIL resulted in a low risk of severe abnormalities during the next screening round, justifying a return to the regular screening programme [34]. Long-term follow-up of test accuracy after implementation of a new test in a prevention programme is of great importance. The Norwegian study illustrates differences in the protective effect of different HPV tests, which underlines the importance balancing clinical sensitivity and specificity (clinical performance) to ensure safety for women participating in screening. It also highlights the importance of ongoing program evaluation when new screening routines are implemented and suggests that further investigation into the question of ASCUS/LSIL triage is needed in Sweden to change policy [34]. Analyses could be completed examining the number of negative follow-up colposcopies that could be avoided and the risk of undetected CIN2+ lesions, by proposed strategy.

A major strength of our study is the linkage to the National Quality Register for Cervical Cancer prevention (NKCx) from which we retrieved all follow-up data and to which all cytological and histological results in Sweden are reported. Correct linkage is ensured by personal identification numbers of all women, which reduces the number of women lost to follow-up. Further, our study reflects a real-life screening setting with women recruited from clinics representing areas of different socio-economic status and the result of long-term clinical follow-up. Since women were recruited directly from the screening program, the results are representative of the population attending screening in Stockholm. We have not adjusted for verification bias. Colposcopy biopsies were taken from visual lesions and women were considered disease free if no lesions were visualised. This could have affected our estimates but it also made our results applicable to other real-life settings. Some women might have been treated because of persistent low-grade disease and therefore censored before development of the outcome. As low and moderate CINs often regress and are less reproducible, the ideal outcome to be chosen would have been CIN3+ or cancer [15,35]. In a clinical setting though, CIN2+ is of great interest as it is the threshold for patient treatment [36].

With regard to study specific considerations, it is important to note that one study reported that most slides diagnosed as ASCUS in the USA and UK is reported as normal in Sweden, which could affect comparisons of results between countries [37]. We have a high prevalence of HPV among our ASCUS and LSIL cases which also could affect the comparability between cumulative risk estimates between countries. Since this study was conducted in the context of the organized screening program and utilized registry-linkages for data collection, no further background information on the study participants was available. HPV testing was completed once in the study making it difficult to speculate whether, and to what extent, new infections occurred during the study.

## Conclusions

In summary, stratifying women with minor cytological abnormalities by HR HPV DNA detection and genotyping helps to identify women at low- and high long-term risk of developing high-grade cervical disease; results that could help inform clinical management. The low risk observed among HPV negatives, given the performance of the HPV test used in the present study, could motivate reconsidering the one-year retest. Specifying the presence of HPV16/18 could focus attention to this high-risk group.
